# Root:shoot ratio in developing seedlings: How seedlings change their allocation in response to seed mass and ambient nutrient supply

**DOI:** 10.1002/ece3.4238

**Published:** 2018-06-22

**Authors:** Tereza Mašková, Tomáš Herben

**Affiliations:** ^1^ Faculty of Science Department of Botany Charles University in Prague Prague Czech Republic

**Keywords:** biomass partitioning, interspecific comparison, nutrient availability, R:S ratio, seed mass, seedling development

## Abstract

Root:shoot (R:S) biomass partitioning is one of the keys to the plants' ability to compensate for limiting resources in the environment and thus to survive and succeed in competition. In adult plants, it can vary in response to many factors, such as nutrient availability in the soil or reserves in the roots from the previous season. The question remains whether, at the interspecific level, reserves in seeds can affect seedlings' R:S ratio in a similar way. Proper allocation to resource‐acquiring organs is enormously important for seedlings and is likely to determine their survival and further success. Therefore, we investigated the effect of seed mass on seedling R:S biomass partitioning and its interaction with nutrient supply in the substrate. We measured seedling biomass partitioning under two different nutrient treatments after 2, 4, 6, and 12 weeks for seventeen species differing in seed mass and covering. We used phylogenetically informed analysis to determine the independent influence of seed mass on seedling biomass partitioning. We found consistently lower R:S ratios in seedlings with higher seed mass. Expectedly, R:S was also lower with higher substrate nutrient supply, but substrate nutrient supply had a bigger effect on R:S ratio for species with higher seed mass. These findings point to the importance of seed reserves for the usage of soil resources. Generally, R:S ratio decreased over time and, similarly to the effect of substrate nutrients, R:S ratio decreased faster for large‐seeded species. We show that the seed mass determines the allocation patterns into new resource‐acquiring organs during seedling development. Large‐seeded species are more flexible in soil nutrient use. It is likely that faster development of shoots provides large‐seeded species with the key advantage in asymmetric above‐ground competition, and that this could constitute one of the selective factors for optimum seed mass.

## 1 INTRODUCTION

Root:shoot (R:S) biomass partitioning is one of the mechanisms by which plants cope with limitations imposed by growth‐constraining resources in the environment (Bloom, 2011; Bonifas & Lindquist, 1990) and may ultimately influence the rate of plant growth (Poorter, 1989). Thus, plants distribute higher proportions of biomass into leaves and stems in nutrient‐rich environments where above‐ground competition for light is strong, whereas in nutrient‐poor environments, where below‐ground competition prevails, they allocate a higher proportion to roots (Tilman, 1985). The relationship between type of competition (above‐ versus below‐ground) and biomass partitioning is not linear, because above‐ground competition is mostly highly asymmetric (Tilman, 1988; Weiner, 1990), in contrast to symmetric or just weakly asymmetric below‐ground competition (Cahill & Casper, 1998; Raynaud & Leadley, 2005). Biomass partitioning in adult plants develops in response to many factors and may show strong lags in these responses (Kobe, Iyer, & Walters, 2010; Mccarthy & Enquist, 2007).

Allocation plasticity is also immensely important for seedlings as the seedling phase is the most vulnerable stage in the generative reproduction cycle for most of plant species and a swift and well‐tuned allocation response can have a direct impact on their survival (Lloret, Casanovas, & Peñuelas, 1999). Seedlings are not able to obtain all their necessary resources from the environment (Deleens, Gregory, & Bourdu, 1999; Nadeem et al., 2013; White & Veneklaas, 2012), and resources stored in the seeds are hence driving force of their early growth (Liu, Siao, & Wang, 2010; Modi & Asanzi, 2008). Importantly, reserves stored in the seed, that is, nonstructural carbon, nitrogen, and phosphorus are fully available to the developing seedling and are highly predictable compared with unpredictable availability of soil nutrients and light.

The ultimate success of a seedling depends on the development of its own resource‐acquiring organs (leaves and roots), but also how well they can respond to the ambient environment, using the predictable maternal resources of all nutrients in the seed (determined primarily by seed mass). Indeed, the proportions of maternally provided and acquired resources change during seedling development. Whereas seedlings invariably develop roots first (to obtain water), their relative investment into leaves versus roots as carbon‐ and nutrient‐acquiring organs, respectively, can also change over time (Gedroc, McConnaughay, & Coleman, 1996; McConnaughay & Coleman, 1999).

Despite different composition, reserves stored in seeds probably can play the same role for seedlings as storage organs do for perennials: allowing the plastic redistribution of the resource, thus supporting optimal biomass allocation in changing conditions (Mironchenko & Kozłowski, 2014). Surprisingly, we know only a little about the role of seed mass in seedling biomass partitioning. Seedling biomass partitioning has been shown to change in time (Nadeem et al., 2013) and response to environmental conditions (Parker, Noland, & Morneault, 2006), but there are no comparative data how it changes among species. Huge interspecific variation of seed mass, and hence amount of stored resources can be used to determine how are these mechanisms, described in one species, working at interspecific level.

The main purpose of this study, therefore, was to distinguish how different nutrient sources (reserves stored in the seeds and nutrients available in the substrate) affect seedling development, particularly biomass allocation, and how it interacts with resource supply from the soil. We hypothesize that (i) each of these two pools of nutrients will have different effects on seedling development, based on the assumption that preferential development of root systems in nutrient‐poor environments occurs to compensate for lack of below‐ground nutrients or to search for nutrient‐rich patches. Furthermore, we specifically hypothesize that: (ii) large seed mass provides enough resources for development and therefore seedlings from larger seeds will be less responsive to substrate nutrient supply during their early ontogeny (first 12 weeks); and (iii) expect that the effect of seed mass on biomass partitioning will decrease with time, whereas the effect of substrate nutrients will increase, with this switch occurring earlier in small‐seeded species.

To distinguish between the impacts of these two pools of nutrients on seedling development, we cultivated seedlings of 17 species—covering a wide range of seed mass—in two different nutrient regimes and measured their biomass allocation during the first 12 weeks of their ontogeny. We examined differences in root and shoot allocation and their changes over time in relationship to differences in seed size and nutrient supply in the substrate. Both at the design and analysis stages, we took into account phylogenetic relationships, because seed mass is strongly phylogenetically conservative trait (Westoby, Leishman, & Lord, 1996) and its effect is likely to be confounded by many other differences when compared naïvely over large phylogenetical distances.

## 2 MATERIALS AND METHODS

### 2.1 Species selection

We selected seventeen common central European eudicot species from nine families, with seed mass ranging over three orders of magnitude (see Table 1), spread regularly over the phylogenetic tree. In all cases but one, for which it was not possible, two species per family were selected, with their seed mass differing as much as possible. Species known to need special treatment to germinate were excluded from the candidate list. All seeds were acquired from a commercial supplier (Planta Naturalis, http://www.plantanaturalis.com). We determined species‐specific seed masses by weighing 100 air‐dried seeds per species (Kleyer et al., 2008). We used this species‐specific seed masses as an approximation of the all resources available to the seedlings. We are aware that this a crude approximation due to possible differences in seed coat or attached structures (such as spines or pappus) between individual species. However, we do not think this is a serious problem in our study given large differences between seed masses of individual species. Furthermore, none of the species we used had attached structures such as spines, except for two species from the Asteraceae family, where both species had a small pappus.

**Table 1:** Species used in the experiment and their seed masses

Species	Avg. per‐seed mass (mg)	Family
*Inula britannica*	7.1	Asteraceae
*Lychnis viscaria*	8.1	Caryophyllaceae
*Sisymbrium officinale*	9.6	Brassicaceae
*Campanula glomerata*	12.2	Campanulaceae
*Campanula trachelium*	17.1	Campanulaceae
*Dianthus deltoides*	18.1	Caryophyllaceae
*Nigella arvensis*	90.1	Ranunculaceae
*Lotus corniculatus*	110.4	Fabaceae
*Ranunculus acris*	114.6	Ranunculaceae
*Plantago lanceolata*	126.5	Plantaginaceae
*Filipendula vulgaris*	160	Rosaceae
*Lithospermum arvense*	161.6	Boraginaceae
*Lepidium campestre*	249.5	Brassicaceae
*Centaurea cyanus*	344.7	Asteraceae
*Anchusa officinalis*	442.1	Boraginaceae
*Lathyrus vernus*	1464.2	Fabaceae
*Agrimonia eupatoria*	1848.5	Rosaceae

### 2.2 Plant cultivation

We used two nutrient treatments ‐ pure deionized water and a universal fertilizer solution (Wuxal Super; manufactured by AGLUKON Specialdünger GmbH & Co.KG, Düsseldorf; N:P:K = 8:8:6) diluted in water to 0.1% concentration. In the experiment, each of these nutrient treatments was used for one half of the seeds and seedlings. The fertilizer concentration used was in the lower half of the range recommended by the fertilizer manufacturer. As substrate, we used expanded perlite (expanded amorphous volcanic glass). We chose perlite because it leaches practically no nutrients, enabling us to fully control the amount of available nutrients by means of our watering and fertilization treatments.

Seeds in both nutrient‐level treatments were germinated individually (to preclude neighbor effects) in Petri dishes on filter paper moistened with 3 ml of the respective fertilizer solution. Deionized water was added throughout germination whenever the filter paper seemed to be almost dry. The Petri dishes were kept in a growth chamber (Adaptis A 1000 with TC kit, Conviron, Canada; light intensity 225 μmol/cm^2^/s at a distance of 12.5 cm from the light source) under the following diurnal temperature regime: 20°C for 12 hr during the day and 10°C for 12 hr during the night. The relative air humidity was set to 50% during the day and 70% during the night. Each seed was transferred into its own individual experimental pot (size 7 × 7 × 8 cm) on the day the radicle emerged through the testa to filter out the effect of that different species differ in their germination lag time. Cultivation of the plants took place in the same growth chamber as that used for germination and with the same temperature, humidity and light settings.

Initially, we aimed to have six replicates per species for each of the two nutrient levels and for each of the four harvesting intervals – after two, four, six and twelve weeks of cultivation. This would have yielded 48 pots per species and 768 pots in total. As not all plants survived transplantations, the real number of replicates per species per nutrient level per harvesting interval ranged from 4 to 6, with the final number of experimental pots 756.

We divided each harvested plant in to their above‐ and below‐ground parts at the boundary between epicotyl and hypocotyl, let them dry at 65°C for 2 days and weighed them to assess their R:S biomass ratios. We did not measure the seed remains at the surface of substrate not connected with seedling itself. We believe that it was composed mainly of structural carbon, which cannot be used by the seedling.

### 2.3 Data analysis

We used two linear mixed‐effect models (LME) with species as a random effect to examine changes in total biomass (sum of root and shoot biomass) and budget of different nutrient sources in total biomass (total biomass:seed mass ratio). Fixed effects included time, nutrient supply and seed mass. We handled phylogenetic relationships of species using phylogenetic eigenvectors (Diniz‐Filho et al. 1998). We used principal coordinate analysis (PCoA; function dudi.pco from the ade4 package version 1.7.4., Dray & Dufour 2007) to extract the first eight eigenvectors from the matrix of phylogenetic distances from the Daphne phylogeny (Durka & Michalski, 2012). These eigenvectors capture 95% of total phylogenetic variation and were used as additional fixed effects in the model. Total biomass, total biomass:seed mass ratio and seed mass were log‐transformed, and the whole model was fitted by maximizing log‐likelihood. We used the *nlme* package version 3.1‐127 (Pinheiro, Bates, DebRoy, & Sarkar, 2016) in R version 3.2.5 (R Development Core Team, 2016). *R* ^2^ was estimated using Nakagawa and Schielzeth's $R_{\mathrm{GLMM}}^{2}$ (Johnson, 2014) as implemented in the rsquared function from R package piecewiseSEM (Lefcheck, 2016).

To determine allometry in R:S biomass partitioning, we used the standard major axis regression, employing *lmodel2* package version 1.7.2 (Legendre, 2014) in R version 3.2.5 (R Development Core Team, 2016) for logarithmically transformed root and shoot biomass.

To analyze the effects of nutrient supply and seed mass on the development of R:S ratio of seedlings over the course of the experiment, we used a linear mixed‐effect model (LME) with species as a random effect. Fixed effects included time, nutrient supply, seed mass and their second and third‐order interactions. We dealt with the phylogenetic relationships of species using the same phylogenetic eigenvectors as in the analysis of total biomass. R:S ratio and seed mass were log‐transformed and the whole model was fitted by maximizing log‐likelihood the same way as in the analysis of total biomass.

When visualizing the analyzed data, we first grouped the species by families to take their phylogenetic relationships into account, and classified species as relatively small‐ or large‐seeded within each family. *Plantago lanceolata,* the single species from the family Plantaginaceae, was treated as a large‐seeded species, because its seed mass is among the largest in this family.

## 3 RESULTS

**Figure 1:**
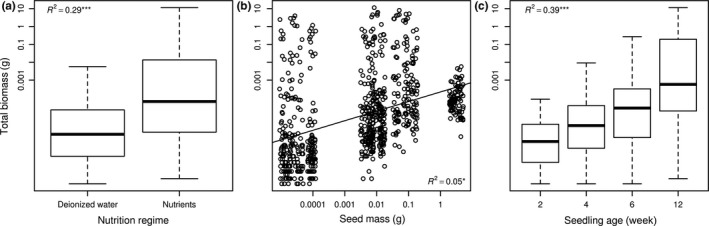
Responses of total biomass to nutrient supply (a), seed mass of species (b) and age of seedling (c). Total biomass was log‐transformed

Total biomass of seedlings increased, unsurprisingly, significantly with nutrient supply (*p* < 0.001, explained variability = 28.9%, see Figure 1a), seed mass (*p* = 0.003, explained variability = 5.3%, see Figure 1b) and time (*p* < 0.001, explained variability = 39.5%, see Figure 1c). Impact of internal seed mass reserves on total biomass (calculated as total biomass:seed mass ratio) decreased significantly with nutrient supply (*p* < 0.001, explained variability = 30.1%) and time (*p* < 0.001, explained variability = 41.2%) and increased with seed mass (*p* < 0.001, explained variability = 5.7%)

We found only a weak signal of allometry in root and shoot biomass (95% confidence interval of slope ranged from 1.02 to 1.08) (Supporting information Appendix S1). For this reason we did not consider allometric relationships between the roots and shoots in the further analyses.

Geometric mean of the R:S ratio varied in deionized water treatement from 0.04 (for *Lathyrus vernus* after 2 weeks of cultivation) to 2.94 (for *Plantago lanceolata* after 12 weeks of cultivation) and in nutrition supply treatement from 0.06 (for *Lathyrus vernus* after 2 weeks of cultivation) to 0.98 (for *Inula britannica* after 12 weeks of cultivation). After 2 weeks of growing, all individual plants in the experiment had developed green cotyledons leaves which allowed them photosynthetic activity.

**Figure 2:**
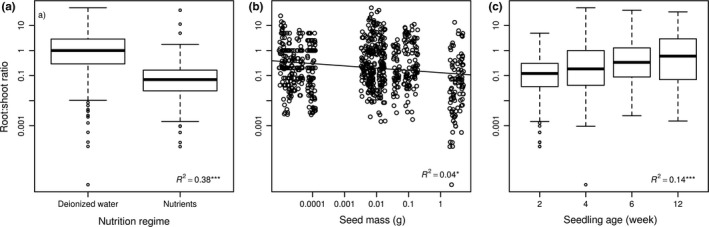
Responses of R:S ratio to nutrient supply (a), seed mass of species (b) and age of seedling (c). R:S ratio was log‐transformed

Seedling R:S biomass partitioning (across plants harvested at all time intervals) was affected both by seed mass and the nutrients available in the substrate. Relative allocation of biomass to roots decreased with amount of available nutrients in the substrate (*p* < 0.001, explained variability = 39.7%, mean R:S ratio decreased from 1.18 in deionized water to 0.37 in the nutrient treatment). Biomass allocation to roots decreased with seed mass of the species (*p* ≤ 0.001, explained variability = 5.7%, mean R:S ratio decreased from 1.27 for the smallest‐seeded species (*Inula britannica*) to 0.57 for the largest‐seeded species (*Agrimonia eupatoria*)) (Figure 2a,b).

**Figure 3:**
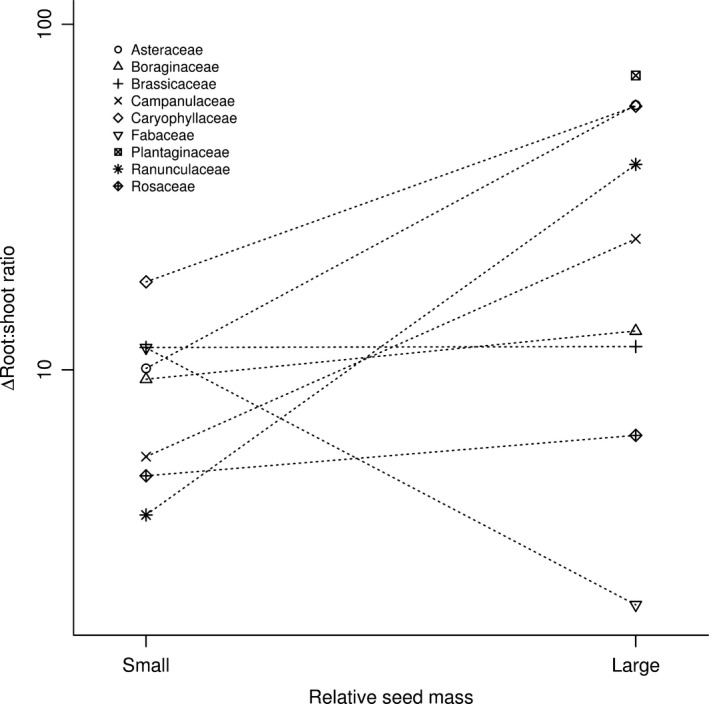
Effects of two independent sources of nutrients (reserves stored in the seeds and nutrients available in the substrate) on seedling development measured as allocation of biomass. Δ R:S ratio was calculated as log(R:S ratio in nutrient supply)/(R:S ratio in deionized water) and it shows difference between R:S ratio in deionized water and R:S ratio in nutrient supply for a given species. Lower values of this difference indicate stronger shift of the R:S ratio in response to increased nutrient supply in the substrate. Higher amount of nutrients available in the substrate translated into higher investment in above‐ground biomass. This trend was stronger for large‐seeded species (with the exception of Fabaceae)

Importantly, the effect of increased nutrient supply on biomass partitioning differed among species. An interaction among nutrient supply and seed mass of species showed that large‐seeded species changed their biomass allocation with changing substrate nutrient supply more than small‐seeded species: namely, they allocated relatively more into their shoots (*p* < 0.001, explained variability = 1.3%) (Figure 3).

Generally, across treatments and species, the proportion of relative biomass allocation to roots increased over time (*p* < 0.001, explained variability = 13.1%, mean R:S ratio increased from 0.5 in the second week to 1.04 in the twelfth week), but in a more detailed view, the time dynamics were changing in relation to both nutrition in the substrate and seed mass. All interactions with time were significant (*p* < 0.001 both for interaction with nutrient supply and seed mass, explained variability = 4.3% and 4.7% for interaction with nutrient supply and seed mass, respectively—see Table 2). The increase in R:S ratio was faster for large‐seeded species and for the treatment without added nutrients.

**Table 2:** Relationship between seedling biomass allocation (measured as R:S ratio) over time, substrate nutrient supply, seed mass and their interaction (linear mixed‐effect model, species used as random effect, model phylogenetically constrained)

Fixed effect	Coefficient	*p* value	*R* ^2^
Time	0.06	<0.001	0.131
Nutrient	−0.25	<0.001	0.397
Seed mass	−1.29	<0.001	0.057
Nutrient*seed mass	0.24	<0.001	0.013
Time*nutrient	−0.03	<0.001	0.043
Time*seedmass	0.13	<0.001	0.047
Time*nutrient*seed mass	−0.02	0.003	0.004

R:S ratio was log‐transformed. Time—age of seedling; nutrient—substrate nutrient supply.

The third‐order interaction among seedling age, nutrient supply and seed mass was significant, but the explained variation was very low (*p* = 0.003, explained variability = 0.4%). The impact of seed mass on biomass allocation in relationship to nutrient supply lasted longer for large‐seeded species (Supporting information Appendix S2).

## 4 DISCUSSION

We found differences between large‐ and small‐seeded species in their biomass partitioning, especially in the way how they respond to nutrient supply across the time. In a striking contrast to our hypothesis, the large‐seeded species were more sensitive to increased nutrient supply. The developmental trajectory over time during the observed initial period of seedling ontogeny also differed between large‐ and small‐seeded species, and it interacted with nutrient supply. Although these interactions had less impact to seedling biomass partitioning than the main effects, we focus on them because the fact that seedlings grow larger over time and in response to higher nutrient supply is well known and is not subject of this work.

**Figure 4:**
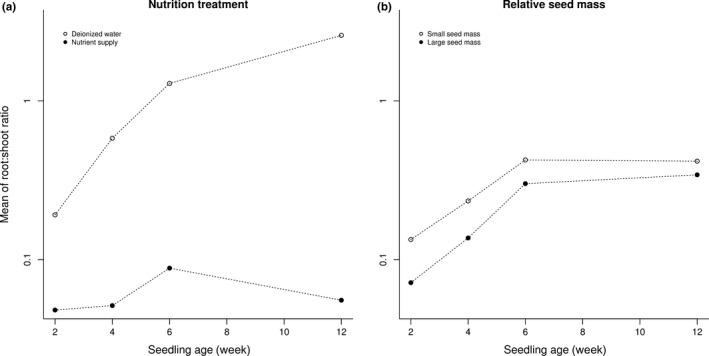
Time courses of seedling biomass allocation. Biomass allocation differed between nutrition regimes and was dependent on relative seed mass

We show, for the first time at the interspecific level, that biomass partitioning is affected not only by soil nutrient supply, but also by the reserves stored in the seeds (seed mass). Our results are generally in agreement with previous studies that documented the relevance of soil nutrient availability for biomass partitioning during different developmental stages of seedlings (Gedroc et al., 1996) and adult plants (Cambui et al., 2011; Glimskär and Ericsson, 1999), but it seems that seed size plays an additional important role in the dynamics of seedling development. Increased amounts of reserves in the seed increased the above‐ground biomass allocation of seedlings. Interestingly, both nutrients sources that we examined, viz. nutrients available in the substrate and reserves stored in the seeds ‐ affected the biomass allocation in a similar way (more available nutrients in whichever pool led to higher investment in shoot biomass ‐ see Figure 4), although they constitute pools with very different dynamics and ecological and evolutionary predictability. Using interspecific comparisons of species differing in their seed mass, we showed that seedling development was not affected only by reserves stored in the seed or nutrients available in the substrate per se, but also by the interaction of these two nutrient sources. This pattern was not an effect of size, because biomass partitioning was measured as the log‐transformed R:S ratio which is essentially size‐independent due to almost complete absence of allometric effects. Contrary to our initial hypothesis, large‐seeded species were more sensitive to increase in substrate nutrient supply than small‐seeded species after phylogenetic correction.

Based on this result, we propose that greater seed size is an adaptation for fast development of shoots, a trait that may be highly beneficial in nutrient‐rich and productive environments. In such environments, fast‐growing shoots may be an important adaptation for success in asymmetric above‐ground competition for light (DeMalach, Zaady, Weiner, & Kadmon, 1990; Morris & Myerscough, 1991), which is supported by the fact that species of productive environments tend to have larger seeds (Herben, Klimešová, & Chytrý, 2017).

In our experiment, we used only successfully recruited seedlings and measured their biomass partitioning after given times. Thus we have information on the impact of nutrient supply on seedling condition, because seedling establishment is often a key part of generative life cycle of plants (Jurena & Archer, 2003; Karban & Thaler, 1999). Large‐seeded species are generally considered to have an advantage during the seedling phase over small‐seeded species, especially in various stressful conditions (reviewed in (Leishman, Wright, Moles, & Westoby, 2000; Westoby, Falster, Moles, Vesk, & Wright, 2002). Nevertheless, most of the previous studies assessed the survival rate of recruited seedlings as a pass‐fail process, counting how many seedlings were surviving after a given time and not examining viability or competitive ability of seedling or saplings later. In contrast to this, we measured seedling R:S ratio as an approximation of competitive ability (which seems to be a good approximation for seedlings which are always much smaller than surrounding adult individuals), although we do not know the direct impact of R:S ratio on fitness in the field.

The changes in biomass partitioning over time differ for each of the nutrient treatments and also for species with different seed masses. Moreover, each of these nutrient pools affected the temporal dynamic of seedling biomass partitioning in different ways (see Figure 4 for comparison of the time dynamics of R:S development for two different pools of available nutrients). Faster increase in the R:S ratio in the treatment without added nutrients was probably caused by relatively rapid and large development of roots searching for nutrient richer patches ‐ this is in agreement with optimal biomass partitioning theory (Bloom, 2011). While such a phenomenon has been reported a number of times (e.g., Portsmuth & Niinemets, 2007; Shipley & Meziane, 2002; Walters & Reich, 2000), there are also contrasting reports showing deviations from this theory's predictions in extreme conditions (Canham, Kobe, Latty, & Chazdon, 2007; Dijkstra & Cheng, 2007; Espeleta & Donovan, 2002).

Our data showed a pattern that conforms with optimal biomass partitioning theory not only in terms of the observed bigger changes in R:S ratio in the nutrient‐poor treatment, but also lower mean of R:S ratio during ontogenetic development for large‐seeded species. Compared to the small‐seeded species, these species probably store more than the minimum necessary reserves in their seeds and thus can dynamically and quickly respond to the environmental conditions into which their seedling emerge. This could be a mechanism that increases probability of seedling survival for large‐seeded species and thus compensates the number of seeds. We assume that this mechanism can be working across the whole gradient of soil fertility under field conditions because we used fairly extreme conditions (pure deionized water and high concentration of nutrient supply) in the experiment. However, the question remains how much is this pattern masked by variation of other environmental factors, such as light, competition with neighbors or composition of microbiota in the soil.

Finally, we would like to draw an analogy between reserves in the seed and reserves stored in roots and rhizomes in adult plants. We hypothesize that seed reserves play a similar role for a seedling as carbohydrate reserves in roots at the beginning of the growing season for an adult plant. The resources stored in the roots during the growing phase are essential at the beginning of the next season just as seed reserves are at the beginning of seedling development (Chapin et al., 1990; Loescher et al., 1990). It remains to be seen whether the effect of nutrients stored in roots/rhizomes has similar effects on dynamics of above‐ground plant parts vs. nutrient‐acquiring parts of their below‐ground structure.

## 5 CONCLUSION

Our study showed that substrate nutrient supply for seedling development is important, but it strongly differs in a nontrivial manner among species. We showed that seedling biomass partitioning during their early ontogeny strongly depends on the resources stored in the seeds (seed mass), and that these resources determine the magnitude of the response to substrate nutrients. Large‐seeded species were more swift in their R:S response to increased substrate nutrients. We also showed that large‐seeded species could have an advantage during the seedling development due to stored resources that are available and ready to use. This also means that the advantage of seed size is mainly in the larger pool of carbon (which is limiting in such environments) and much less in the larger pool of soil‐borne resources, such as nitrogen or phosphorus. Thanks to these stored resources, large‐seeded species are more flexible, can effectively and quickly use nutrients available in the soil and develop shoots faster—which will provide them the key advantage in asymmetric above‐ground competition. This could constitute a selective factor for optimum seed mass, namely under highly productive conditions.

## AUTHOR CONTRIBUTION

TM and TH designed the experiment, TM performed the experiment, TM and TH analyzed and interpreted results, TM wrote the text with contributions of TH. Both authors approved the final version of the manuscript.

## DATA ACCESSIBILITY

All measured data are available as a Dryad item [will be submitted upon acceptance] at https://datadryad.org.
