# Supplementary figures and images for: Root:shoot ratio in developing seedlings: How seedlings change their allocation in response to seed mass and ambient nutrient supply
Source: Ecol Evol. 2018 Jun 22;8(14):7143–50. doi: 10.1002/ece3.4238 (PMC6065327; doi:10.1002/ece3.4238)

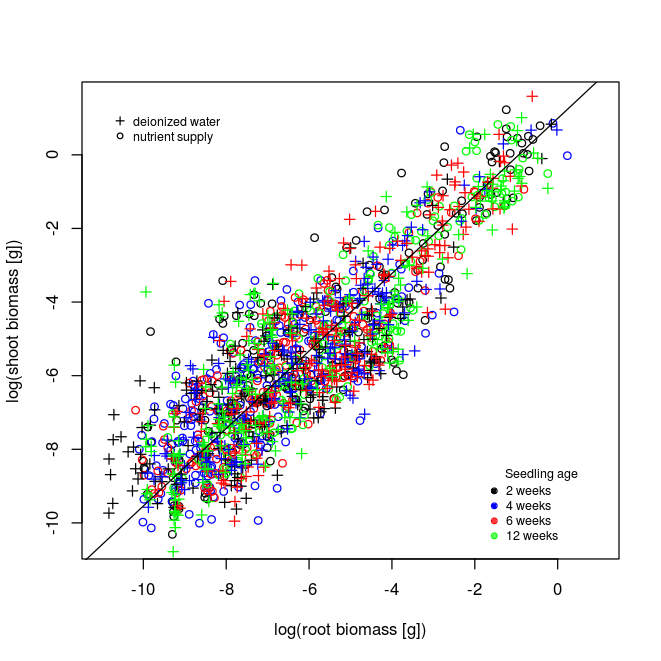

Supplement: Supplementary file 1 [file ECE3-8-7143-s001.tiff]

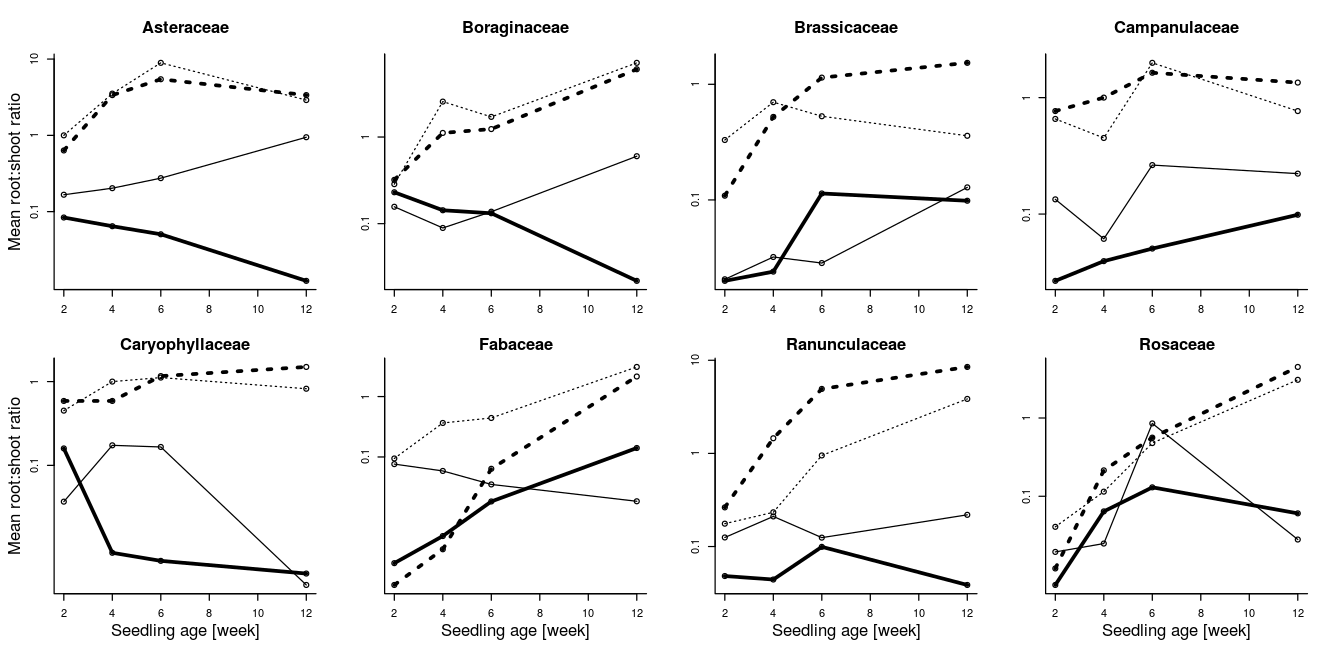

Supplement: Supplementary file 2 [file ECE3-8-7143-s002.tiff]
